# Mixed‐Methods Study Identifying Key Intervention Targets to Improve Participation in Daily Living Activities in Primary Sjögren's Syndrome Patients

**DOI:** 10.1002/acr.23536

**Published:** 2018-05-21

**Authors:** Katie L. Hackett, Katherine H. O. Deane, Julia L. Newton, Vincent Deary, Simon J. Bowman, Tim Rapley, Wan‐Fai Ng, United Kingdom Primary Sjögren's Syndrome Registry, Sue Brailsford, Joanne Dasgin, Theodoros Dimitroulas, Lucy Kadiki, Daljit Kaur, George Kitas, Esther Gordon, Cathy Lawson, Gill Ortiz, Elizabeth Price, Suzannah Pegler, Gavin Clunie, Suzanne Lane, Ginny Rose, Sue Cuckow, Bridget Griffiths, Sheryl Mitchell, Christine Downie, Sarah Legg, Susan Pugmire, Saravanan Vadivelu, Anne Field, Stephen Kaye, Devesh Mewar, Patricia Medcalf, Pamela Tomlinson, Debbie Whiteside, Neil McHugh, John Pauling, Julie James, Andrea Dowden, David Coady, Elizabeth Kidd, Lynne Palmer, Bhaskar Dasgupta, Victoria Katsande, Pamela Long, Olivia Godia, Usha Chandra, Kirsten MacKay

**Affiliations:** ^1^ Newcastle University Newcastle upon Tyne Hospitals National Health Service Foundation Trust, and Northumbria University Newcastle upon Tyne UK; ^2^ University of East Anglia Norwich UK; ^3^ Newcastle University and Newcastle upon Tyne Hospitals National Health Service Foundation Trust Newcastle upon Tyne UK; ^4^ Newcastle upon Tyne Hospitals National Health Service Foundation Trust and Northumbria University Newcastle upon Tyne UK; ^5^ Queen Elizabeth Hospital Birmingham UK; ^6^ Newcastle University and Northumbria University Newcastle upon Tyne UK

## Abstract

**Objective:**

Functional ability and participation in life situations are compromised in many primary Sjögren's syndrome (SS) patients. This study aimed to identify the key barriers and priorities to participation in daily living activities, in order to develop potential future interventions.

**Methods:**

Group concept mapping, a semiquantitative, mixed‐methods approach was used to identify and structure ideas from UK primary SS patients, adult household members living with a primary SS patient, and health care professionals. Brainstorming generated ideas, which were summarized into a final set of statements. Participants individually arranged these statements into themes and rated each statement for importance. Multidimensional scaling and hierarchical cluster analysis were applied to sorted and rated data to produce visual representations of the ideas (concept maps), enabling identification of agreed priority areas for interventions.

**Results:**

A total of 121 patients, 43 adult household members, and 67 health care professionals took part. In sum, 463 ideas were distilled down to 94 statements. These statements were grouped into 7 clusters: Patient Empowerment, Symptoms, Wellbeing, Access and Coordination of Health Care, Knowledge and Support, Public Awareness and Support, and Friends and Family. Patient Empowerment and Symptoms were rated as priority conceptual themes. Important statements within priority clusters indicate patients should be taken seriously and supported to self‐manage symptoms of oral and ocular dryness, fatigue, pain, and poor sleep.

**Conclusion:**

Our data highlighted the fact that in addition to managing primary SS symptoms, interventions aiming to improve patient empowerment, general wellbeing, access to health care, patient education, and social support are important to facilitate improved participation in daily living activities.

## Introduction

Primary Sjögren's syndrome (SS) is a systemic autoimmune disease characterized by sicca symptoms 1. Additionally, extraglandular symptoms are commonly experienced 2, including pain 3, sleep disturbances 4, fatigue 5, low mood, and anxiety 6. These symptoms impact significantly on quality of life 7, 8, and many patients experience difficulty with participation (involvement in a life situation) and undertaking functional activities 9, 10. Examples include problems with hygiene, grip, reach, eating, transfers, mobility, vocational activities, and sexual activity 9, 10, 11, 12. There are currently no effective disease‐modifying treatments available, and management strategies typically focus on symptom management. Pharmacologic treatments mainly comprise topical treatments for dryness as well as systemic treatments 13, 14. However, such treatments have limited effect on the quality of life 15, 16, 17.

**Significance & Innovations**

- Widespread stakeholder engagement with patients, family, and health care professionals has identified key priority themes, including Patient Empowerment, Symptoms, Wellbeing, and Access and Coordination of Health Care, which can all be addressed to improve functional ability in primary Sjögren's syndrome patients.
- The greatest priority is to take primary Sjögren's syndrome patients seriously and to provide individualized support to self‐manage the symptoms of dryness, fatigue, pain, and poor sleep.

Previous studies have demonstrated an association between functional impairment, disease activity, pain, and fatigue 9, 12. However, the key barriers to the performance of daily function and participation among primary SS patients have not been systematically studied. In addition to gathering information from primary SS patients, close family members can often provide useful insight into factors that interfere with the daily activities of the patients. Furthermore, in planning future interventions that are effective and feasible to improve daily function and participation of primary SS patients 18, 19, it is important to understand the perspective of both potential users (primary SS patients), their supporters (people they live with), and the health care providers who are likely to deliver the interventions in the future. To our knowledge, there have been no published studies investigating perspectives of other stakeholder groups such as family members and health care professionals (HCPs) who provide care to primary SS patients. The aim of the study was to identify key barriers and priorities to participation in daily living activities for primary SS patients, as targets for future interventions.

## Subjects and methods

**Figure 1:**
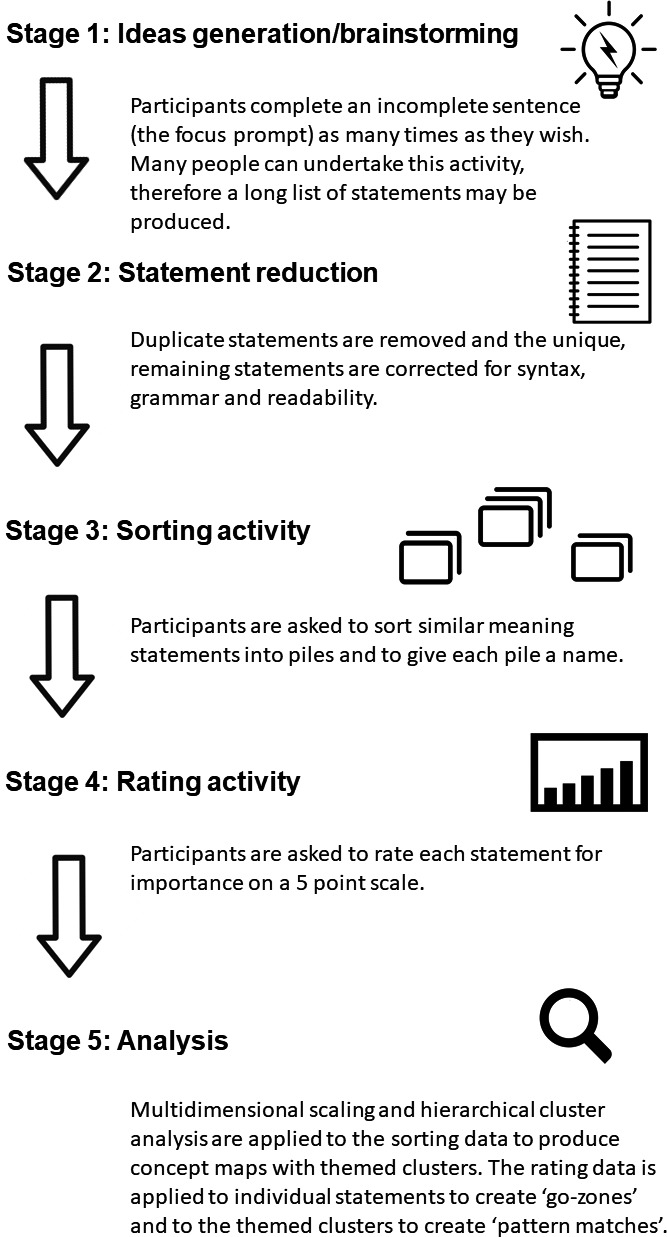
The 5 stages of group concept mapping.

We used group concept mapping (GCM) methodology 19 to determine important key barriers to participation and daily function in primary SS patients (Figure 1). Our specific objectives were to identify barriers/facilitators to participation and performance of daily activities, structure the generated ideas into clusters or themes through a sorting exercise, identify priority targets from the identified barriers/facilitators and themed clusters, and compare similarities and differences in priorities between different stakeholder groups.

GCM has been used in the rheumatic diseases to investigate treatment for hip and knee osteoarthritis 20, to design and develop online self‐management interventions 21, and in a program to prevent work disability in rheumatoid arthritis patients 22. We have previously used this approach to evaluate and plan improvements to a fatigue service 23. The advantage of using this mixed‐methods approach over qualitative interviews or focus groups is that it mixes both qualitative and quantitative methods, large numbers of stakeholders can be consulted, and it provides a consensus vision containing the prioritized ideas of all participants.

GCM is a semiquantitative, mixed‐methods participatory approach, which uses a combination of individual and group processes (brainstorming, sorting, rating, and interpretation) and multivariate statistical analyses (multidimensional scaling and hierarchical cluster analysis). These activities result in visual depictions of all stakeholders’ opinions in the form of concept maps. Participants add quantitative values to qualitative statements that are gathered during the brainstorming phase, and these statements can be interpreted in pattern matches and go‐zones and used in planning or evaluation studies 24. We published the methods of this study a priori 25.

### Participant groups

We recruited participants from 3 stakeholder groups. First, we recruited primary SS patients (ages ≥18 years) who were members of the United Kingdom Sjögren's Syndrome Registry (UKPSSR) 26 and who therefore fulfilled the American‐European Consensus Group classification criteria 27. The second group comprised adults living with a primary SS patient (adult household member [AHM]). The final group was HCPs working with primary SS patients. We were granted approval to recruit UKPSSR participants from 12 different sites in England. We invited all UKPSSR participants at these sites to take part via a mailed written invitation. An enclosed invitation was included in the pack addressed to an AHM.

### Data collection and analysis

We collected baseline age and sex demographics from primary SS and AHM participants. Primary SS participants also indicated whether they received disability benefits, the number of dependents living with them, and their employment status, and they completed a range of symptom scores 28, 29, 30. We asked HCPs to specify which professional group they belonged to and complete a measure of caregiver strain 31. This GCM study took place in 5 discrete stages, and we gave participants the option to complete the activities either online or on paper. Participants could also choose to complete the brainstorming at a face‐to‐face meeting at Newcastle.

#### Stage 1: ideas generation/brainstorming

We asked potential participants to respond to a focus prompt, an incomplete sentence that they could complete as many times as they wished. The research team designed the focus prompt, and it went through several iterations. The precise wording of the focus prompt aimed to capture barriers and facilitators to participation in daily activities for people with primary SS, using lay terms. The focus prompt was: “People with Sjögren's could do more of the things they want to do and have to do if…”

This process generated a list of statements/ideas from all participants taking part in this stage of the study. A participant completing this exercise online could see statements provided by other participants who had completed the brainstorming activity previously. We added statements received by participants in both the face‐to‐face meeting and from postal replies to the online interface. Therefore, participants taking part online could also see the statements provided through the alternative data collection methods. Brainstorming was continued until data saturation was achieved 19, 24, at which point no further unique ideas were being generated through the brainstormed responses 32.

#### Stage 2: statement reduction

In this second stage, the full list of statements was reduced to a shorter list of unique ideas by several members of the research team (KLH, VD, and TR). First, we split statements containing more than one idea into separate statements. Next, we applied a key word to each statement, formed groups of statements containing the same key word, and considered them in turn. We removed duplicate statements and combined those that described the same or overlapping idea 24. Subsequently, the refined statement list was reviewed for syntax and readability by the research team, 2 patients with primary SS, and an AHM.

#### Stage 3: sorting activity

During the statement reduction process, similar statements were considered together. Applying a random number (i.e., 1–94) to each statement, in effect, shuffled the statement list prior to the sorting activity. The statements were numbered and randomized within the software used for this GCM project (CS Global MAX). The numbered statements were printed onto individual cards and participants were asked to sort them by creating piles of similar meaning statements. They were asked to name each pile and to record the name of each name and numbers of the statements contained within each pile. Those opting to take part online could sort statements into virtual piles.

#### Stage 4: rating activity

Participants were given a list of the numbered statements and asked to rate them for importance on a 1–5 Likert scale (where 1 = relatively unimportant and 5 = extremely important).

#### Stage 5: data analysis

Sorting and rating data were analyzed in the CS Global MAX software. Multidimensional scaling was applied to the sort data, which were arranged into a similarity matrix to position each statement in relation to others as a point on an X‐Y axis. This arrangement results in a 2‐dimensional representation of the statements, and each statement is represented by a numbered point on a map. Multidimensional scaling produces a stress value that indicates the goodness‐of‐fit of the map with the raw data and stability within the overall map. A stress value below 0.36 is preferred in concept mapping studies 33. Statements that were frequently sorted together end up being closely located near each other on the map, as participants considered them to be similar conceptually during the sorting activity. Hierarchical cluster analysis (Ward's method) was next applied to the data, resulting in clusters of statements, which were examined by the authors, who agreed on an overall cluster solution through discussion. The software suggests labels for clusters based on the names participants give to their piles during the sorting exercise and appropriate cluster names were selected using these suggestions.

Importance ratings were considered at cluster level in a pattern match, which demonstrates differences between the importance ratings attributed by each participant group to the clusters. Importance ratings were also considered at statement level in go‐zones. These are scatter plots comparing importance ratings for each statement within a cluster for 2 groups. To make a visual 2‐group comparison, groups with both the lived experience of primary SS (patients and AHMs) were combined and compared with the HCP group. A statement falling within the top right quadrant of the go‐zone (demarcated by the mean importance ratings for each group) indicates it is a priority for both lived experience and HCP groups. Go‐zones were generated for each cluster.

### Ethics approval

Ethical approval was granted by the Office for Research Ethics Committees Northern Ireland (13/NI/0190, IRAS Ref: 125562), and the study was registered on the National Institute of Health Research Comprehensive Clinical Research Network's portfolio of noncommercial studies (study ID: 15939).

## Results

### Participant characteristics

From the 371 patients invited to participate in the study, 49% replied that they would like to take part, and 33% of patients completed 1 or more stages of the GCM exercise. Flow diagrams of participants through the study can be seen in Supplementary Figure 1, available on the *Arthritis Care & Research* web site at http://onlinelibrary.wiley.com/doi/10.1002/acr.23536/abstract. In total, 231 participants took part, including 121 primary SS patients, 43 AHMs, and 67 HCPs. The mean ± SD age of patient participants was 63 ± 10 years, and 64 ± 9.5 years for AHMs. Descriptive statistics demonstrating demographic data for both primary SS and AHM participants and patient symptom scores can be seen in Table 1.

**Table 1:** Demographic information and symptom scores for patients and demographics and Caregiver Strain Index scores for adult household members^a^

Characteristics and measurements	Values
Primary Sjögren's syndrome patients	
Age, mean ± SD years	63.01 ± 9.96
Years since diagnosis, mean ± SD	10.15 ± 7.21
Female, %	87
Live with another adult, %	73.50
Dependents living at home, %	18
Employment, %	
Unemployed	5.7
Employed part‐time	17
Employed full‐time	14.8
Housewife/husband	46
Retired	57.9
Receiving disability benefits, %^b^	22
HADS anxiety (range 0–21)	7 (6)
HADS depression (range 0–21)	6 (5.7)
Pain VAS (range 0–100)	37.3 (27.4)
Fatigue VAS (range 0–100)	54.6 (29.2)
Mental fatigue VAS (range 0–100)	38.1 (28.7)
Dryness VAS (range 0–100)	56.7 (30)
Cognitive Failures Questionnaire (range 0–100)	43.2 (18)
Improved Health Assessment Questionnaire (range 0–100)	17.2 (36.7)
Adult household members	
Female, %	37.2
Age, mean ± SD years	62.7 ± 11.4
Years since diagnosis of household member, mean ± SD	10.7 ± 7.9
Caregiver Strain Index (range 0–13)	1 (3)

a Scores reported as median (interquartile range) unless otherwise indicated. HADS = Hospital Anxiety and Depression Scale; VAS = visual analog scale.

b Disability Living Allowance, Attendance Allowance, Personal Independent Payments, Independent Living Fund, Employment and Support Allowance or Incapacity Benefit, Caregiver Strain Index.

The HCP group included doctors (hospital doctors and general practitioners), therapists (physiotherapists, occupational therapists, psychologists, and podiatrists), nurses, and a service manager. A breakdown of professional groups within the HCP participants can be seen in Supplementary Figure 2, available on the *Arthritis Care & Research* web site at http://onlinelibrary.wiley.com/doi/10.1002/acr.23536/abstract.

### Statements from stages 1 and 2 and concept maps generated from stages 3, 4, and 5

**Figure 2:**
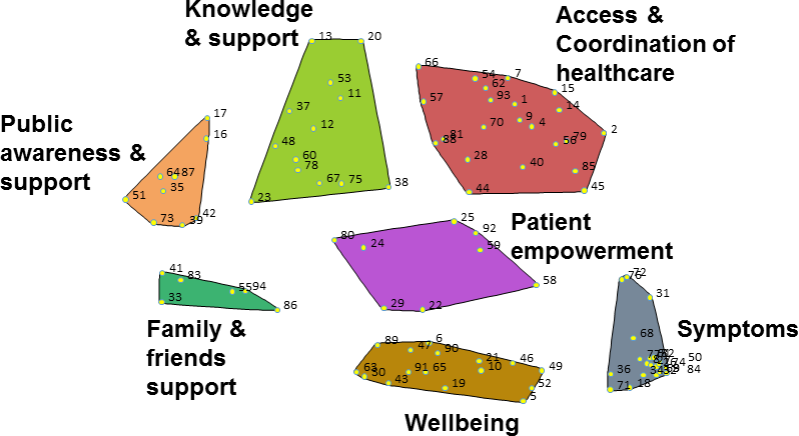
Point cluster map showing the 7 themed clusters. Color figure can be viewed in the online issue, which is available at http://onlinelibrary.wiley.com/doi/10.1002/acr.23536/abstract.

Brainstorming generated 463 statements, which were distilled to a final set of 94 unique statements. Multidimensional scaling resulted in a point map with a stress value of 0.18. A 7‐cluster solution was agreed upon and contained the following named clusters: Access and Coordination of Health Care, Knowledge and Support, Public Awareness and Support, Friends and Family, Symptoms, Patient Empowerment, and Wellbeing. The smallest cluster (Friends and Family) contained 6 statements and the largest (Access and Coordination of Health Care), 22 statements. The point cluster map is shown in Figure 2.

Statements belonging to the Patient Empowerment cluster received the highest priority ratings of a possible 5, with a mean ± SD of 4.07 ± 0.24, followed by the Symptoms cluster with a mean ± SD importance rating of 3.98 ± 0.33 for each statement. The next most important clusters were Wellbeing (mean ± SD 3.91 ± 0.38), Access and Coordination of Health Care (mean ± SD 3.89 ± 0.43), Knowledge and Support (mean ± SD 3.74 ± 0.39), and Friends and Family (mean ± SD 3.69 ± 0.30). The lowest rated cluster was Public Awareness and Support (mean ± SD 3.48 ± 0.36). Mean importance scores for each of the themed clusters, together with the mean rating scores for the individual statements within them can be viewed in Table 2.

**Table 2:** Mean importance ratings for the clusters and each of the numbered statements within each cluster

Statements and clusters	Importance (1–5)
Cluster 1: Patient Empowerment	4.07
29. There was a cure	4.45
80. Felt they were being taken seriously	4.34
25. Have support to manage their symptoms themselves	4.22
58. Take their medication as prescribed	4.07
24. Have confidence to seek advice when needed	4.02
22. Look after their physical, emotional, and spiritual wellbeing	4.00
92. Were taught relaxation techniques	3.74
59. Have support with memory and concentration difficulties	3.73
Cluster 2: Symptoms	3.98
84. Their eyes were less dry	4.37
50. Have less pain	4.34
18. Were less fatigued	4.34
76. Fatigue was better managed/treated	4.34
32. Their vision was not impaired	4.32
61. Their eyes were more comfortable	4.31
71. Were able to sleep better	4.23
68. Have healthy teeth and/or comfortable dentures	4.17
69. Their throat was less dry	4.16
34. Swallowing was easier	4.10
82. Mouth and lips were less dry	4.07
72. Gastrointestinal (stomach and bowel) problems were managed	3.93
8. Were less prone to getting infections	3.89
31. Skin problems were treated	3.76
74. Were less breathless	3.64
77. Have more feeling in their mouth and lips	3.64
3. Did not have mouth sores or ulcers	3.61
36. Did not have sexual problems	3.54
27. Their skin was less dry	3.46
26. Their vagina was less dry	3.40
Cluster 3: Wellbeing	3.91
91. Keep their mind active	4.41
43. Have a positive attitude	4.41
90. Keep their body active	4.36
21. Feel in control of their symptoms	4.24
89. Develop good coping strategies	4.17
47. Exercise regularly	4.01
65. Learn to balance their activity and rest	3.96
46. They have better mobility	3.90
19. Could come to terms with their symptoms	3.84
6. They have a good diet	3.79
63. Could come to terms with their limitations	3.78
49. Could improve their concentration	3.75
52. Their mood was better	3.73
10. Were less stressed or worried	3.69
30. Could continue to drive	3.68
5. Could go out in the sun	2.86
Cluster 4: Access and Coordination of Health Care	3.89
54. There is good communication between clinicians	4.45
2. Have access to a range of good drug treatments	4.39
14. Have professional support during a flare of symptoms	4.37
1. Know who to contact when they have a flare of symptoms	4.32
85. Associated conditions are diagnosed and treated	4.31
15. Can see a consultant when needed	4.25
93. Their health care is better coordinated	4.20
4. Knew the range of available treatment options	4.18
79. Diagnosis was quick	4.17
66. There was more funding for specialist rheumatology services	4.12
7. There were one‐stop Sjögren's clinics with all relevant health care professionals available	4.02
62. Have access to a specialist nurse	3.91
40. Have an individualized treatment plan	3.90
45. There was better management of the side effects of drugs	3.88
56. Health care professionals would raise sensitive topics (e.g., sex and vaginal dryness) during consultations	3.71
57. Professionals could direct them to support groups and charities	3.66
28. Have access to psychological support	3.49
70. Have access to occupational therapy	3.46
9. Have access to physiotherapy	3.39
88. Have access to complementary therapies or alternative remedies	3.23
44. There were diaries for recording symptoms and problems to bring to appointments with health care professionals	3.16
81. Have access to hydrotherapy	2.96
Cluster 5: Knowledge and Support	3.74
20. There was more good research to test and develop treatments	4.45
13. There was more good research to understand the underlying causes	4.38
53. There was education on Sjögren's for health care professionals	4.28
37. There was education on Sjögren's for patients	3.97
75. There was information available on exercise and Sjögren's	3.67
60. Have access to support and advice from other people with Sjögren's	3.66
78. Have help with dental costs	3.65
67. Felt a family member or supporter would be welcome at their appointments	3.55
48. Have access to appropriate aids and adaptations in their homes	3.51
23. Felt a family member or supporter could be included in their care planning	3.48
11. Have Sjögren's advice leaflets	3.44
38. Could access support to help set personal goals	3.44
12. There were appropriate aids and adaptations in the community	3.17
Cluster 6: Friends and Family	3.69
41. Have supportive family and friends	4.12
83. Family could understand the symptoms	3.83
94. Can explain to others what they can and cannot do	3.78
55. Could easily describe Sjögren's to others	3.66
33. Friends and family include them in events	3.65
86. On a bad day people could tell by looking at them how they are feeling	3.10
Cluster 7: Public Awareness and Support	3.48
17. There was education about Sjögren's for people who fund services	4.01
87. Those unable to work and/or who needed support to function were eligible for benefits	3.92
64. Employers were aware of things they could do in the workplace that are helpful for people with Sjögren's	3.85
51. There was education on Sjögren's for family members	3.59
16. There was education about Sjögren's for the general public	3.38
35. Public spaces were more Sjögren's friendly, e.g., heated/lit/air conditioned differently	3.28
39. Have a disabled parking badge	3.14
73. Public transport was accessible	3.09
42. Have assistance with shopping, cleaning, etc.	3.04

**Figure 3:**
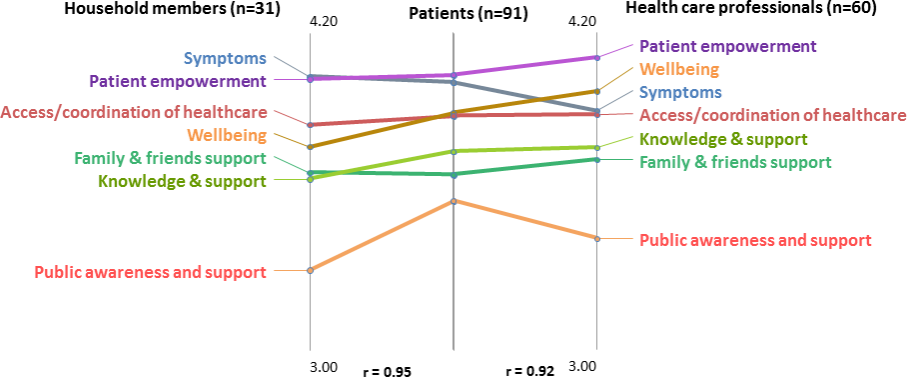
Pattern match depicting the mean importance ratings by participant group.

**Figure 4:**
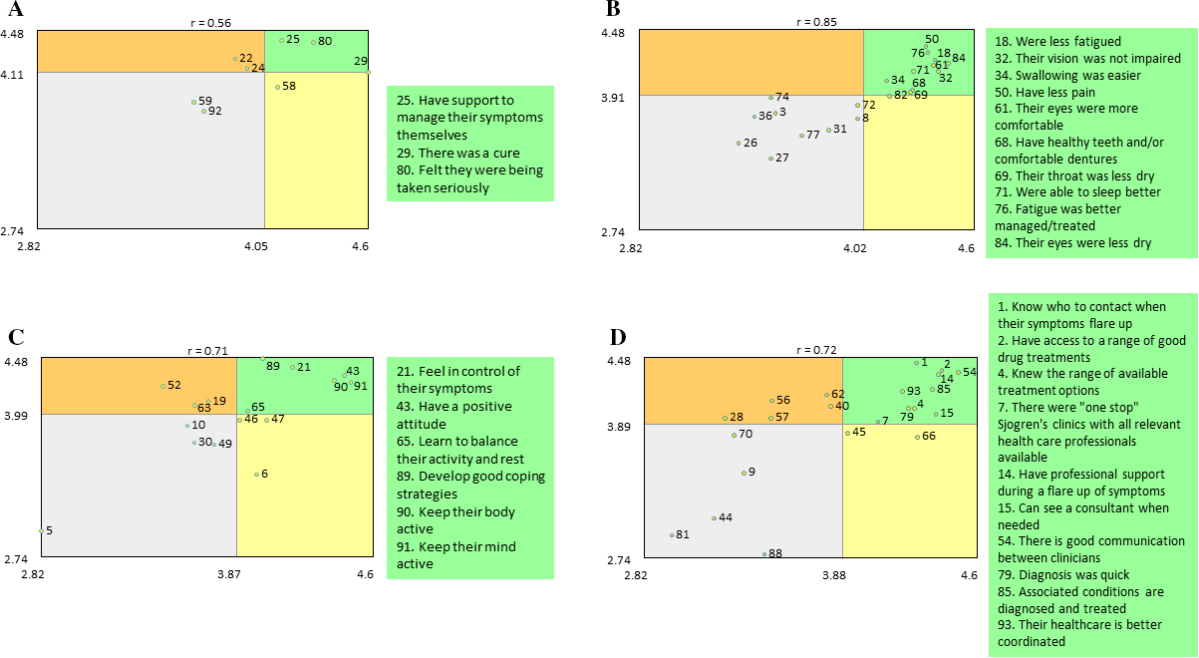
Go‐zones showing the most important statements within the most important clusters. **A**, Patient empowerment; **B**, Symptoms; **C**, Wellbeing; **D**, Access and coordination of health care. For all 4 zones, health care staff: n = 61, and patients and household members: n = 124.

Average importance rating scores for each cluster have been broken down by stakeholder group and are shown in Figure 3. Importance is rated 1–5, with 5 being the maximum possible score. Go‐zones demonstrating the most important statements within the clusters as determined by all participants, as agreed by both health care staff and the combined primary SS patient and household groups, are shown in Figure 4. Priority statements are presented in the upper right quadrants of each go‐zone, which are demarked with mean importance scores for each cluster. The remaining go‐zones can be seen in Supplementary Figure 3, available on the *Arthritis Care & Research* web site at http://onlinelibrary.wiley.com/doi/10.1002/acr.23536/abstract.

#### Patient Empowerment (8 statements)

This cluster was rated the most important cluster by both the primary SS and AHM group. Within this cluster, the statements #80 “Felt they were being taken seriously,” #25 “Have support to manage their symptoms themselves,” and #29 “There was a cure” all fell within the top right priority area of the go‐zone.

#### Symptoms (20 statements)

Statements within this cluster all related to symptoms of primary SS. Statements within the priority go‐zone quadrant all related to symptoms of fatigue, sleep disturbances, pain, and oral or ocular dryness.

#### Wellbeing (16 statements)

Statements falling within the priority quadrant include #21 “Feel in control of their symptoms,” #43 “Have a positive attitude,” #65 “Learn to balance their activity and rest,” #89 “Develop good coping strategies,” and keeping both mind and body active (#90 and #91). There was some disagreement regarding the priorities of other statements within this cluster. The HCP group perceived statement #19 “Could come to terms with their symptoms,” #63 “Could come to terms with their limitations,” and #52 “Their mood was better” as being priority statements, whereas those with the lived experience did not. Those in the lived experience group rated a good diet, better mobility, and regular exercise as priorities, whereas the HCP group regarded these concepts as being less important.

#### Access and Coordination of Health Care (22 statements)

Statement #54 “There is good communication between clinicians” was rated as the most important within this cluster (Table 2). Other priority statements indicate that one‐stop clinics where a range of HCPs can be seen in 1 visit (#7), professional support during flares of the condition (#14), clarity about who can be contacted if symptoms do flare up (#1), and access to a consultant if required (#15) were all deemed as being important factors.

#### Knowledge and Support (13 statements)

The most important statements within this cluster were those relating to the need for more research to develop and test treatments and research to understand the causes of the disease. These were closely followed by statements relating to education on primary SS for both HCPs and patients.

#### Friends and Family (6 statements)

The most important statement within this cluster was #41 “Have supportive family and friends.” It was also deemed important that family could understand primary SS symptoms (#83) and were able to explain to others what they could or could not do (#94).

## Discussion

This study has identified factors that stakeholders have deemed to interfere with performance of the daily activities and participation in people with primary SS. These factors have been grouped into conceptual cluster themes through the sorting process undertaken by stakeholders and subsequent multiple dimensional scaling and cluster analysis. The stress value of the point map generated during the multidimensional scaling process was low (0.18), demonstrating stability within the concept map (ideal stress values should fall below 0.39) 19. The factors were also prioritized at cluster level, with individual priority factors identified within each cluster through ascertaining the mean rating scores of the individual statements within each of the clusters.

To our knowledge, only 1 published study has explored patients’ viewpoints on determinants they consider to interfere with their performance of daily activities and quality of life 34. In this qualitative focus group study, the authors found 3 broad domains containing 86 concepts. These domains were the physical dimension, psychological and emotional challenges, and social life and daily living. The most commonly reported factors were from the physical domain, and specific factors within this domain included pain, dryness, shortness of breath and constipation 34. However, only a small number of patients were involved (n = 20), consequently, additional concepts may have been missed. Moreover, the authors did not use a structured approach to identify priority factors for intervention and did not seek the perspectives of close family members of the patients and health care providers. Our study has addressed these limitations. Indeed, our data have identified additional concepts not previously reported, such as access to and coordination of health care.

The primary SS participants who took part in this study had a mean age of 63 years, similar to the average age of UKPSSR participants (61 years) 7, which indicates that our sample was representative of this cohort. The average number of years since diagnosis in this study was 10. Conceivably, newly diagnosed primary SS patients may have different needs, and a separate study is required to address this possibility.

Our data show that the most important themed cluster was Patient Empowerment. Patient empowerment is a process where people improve their capacity to use their own resources to navigate their health care and live well with their chronic conditions 35. Others have described the potential for patient empowerment as occurring at 3 levels: at a patient level (e.g., patients’ rights, responsibilities, and opportunities), a health care provider level (e.g., through individual focused empowering intervention), and at a health care system level (e.g., provision of group empowering intervention) 36. The Patient Empowerment cluster was located centrally in the map, indicating a connection between this themed cluster and the surrounding clusters. Priority statements within the Patient Empowerment cluster demonstrate that in order to empower patients, HCPs need to take them seriously (#80) and support them to manage their symptoms themselves (#25). Multidisciplinary education has been shown to empower rheumatoid arthritis patients to manage their condition and reduce disease activity in the longer term 37. We hypothesize that addressing these factors, for example through individualized interventions supporting patients to manage their oral and ocular dryness symptoms and with nonpharmacologic interventions such as exercise 38, 39, pain management 40, 41, and cognitive behavioral therapy for sleep disturbances 4, patients may feel more patient empowered. Modes of delivering these interventions need to be considered, and digital technologies, such as the use of mobile applications, can be used to empower patients to take charge of their own health 42 and used as an adjunct to face‐to‐face care.

The go‐zone statements within the Wellbeing cluster include potential facilitators to self‐managing symptoms such as fatigue and pain, including balancing activities and rest (#65) and developing good coping strategies (#89). These could be incorporated into a complex nonpharmacologic behavior‐change intervention package and ultimately empower patients to self‐manage their symptoms 36. Within the Wellbeing cluster, there was also some disagreement between the HCP and lived experience groups. HCPs considered mood as being an important factor, whereas the lived experience group did not prioritize this concern. Other studies have demonstrated a relationship between mood and quality of life 7, fatigue 43, and pain 44. However, these studies have not been able to determine whether mood is a consequence of these symptoms or a causal factor. Our study shows that although HCPs regard mood as a priority, patients prioritized other symptoms first. This might be due to patients viewing their low mood as being a consequence of these symptoms 34.

Our data also suggest that addressing the structure of health care systems and how patients can access them could influence patients’ ability to function better. For instance, primary SS patients may see different specialists because of the diverse symptomatology of the disease. Allowing patients’ access to several specialists within a single clinic would improve patient access and facilitate communication between clinicians.

Our data are presented as priorities within each go‐zone, and these may be helpful when designing services and interventions for primary SS patients. However, it is important to stress that individual patients have different priorities, and a personalized approach is essential. To provide a personalized approach, holistic and multidisciplinary care is required. Embedding access to multidisciplinary support within clinical services (and addressing the priority statements within the Access and Coordination of Health Care cluster) would facilitate individualized care.

This study is not without limitations. Only 33% of patient participants invited to take part in the study went on to complete one or more stages of the GCM activities. We therefore cannot rule out possible selection bias. However, the mean age of participants in this study (63 years) is similar to the age of a recent study which included the majority of the UKPSSR cohort (mean age 61 years) 7. Secondly, 13% of the primary SS patients who took part in this study were male, which is slightly greater than the proportion of males (9%) reported in a recent meta‐analysis of primary SS studies that included 7,888 participants 45. We therefore compared differences in importance ratings for each cluster between male and female primary SS participants (males n = 10, females n = 83) by generating a further pattern match, which revealed no differences in importance ratings between males and females (r = 0.99). Therefore, despite a relatively greater proportion of male primary SS patients taking part in this study, there was a very high level of agreement between males and females, and the increased male representation did not influence the overall priority scores.

In conclusion, our study has identified several key areas as targets for planning future interventions to support improvements in daily function and participation in primary SS patients. Empowering patients by taking their health concerns seriously and supporting them to self‐manage their condition is the greatest priority.

## Author contributions

All authors were involved in drafting the article or revising it critically for important intellectual content, and all authors approved the final version to be submitted for publication. Dr. Hackett had full access to all of the data in the study and takes responsibility for the integrity of the data and the accuracy of the data analysis.

### Study conception and design

Hackett, Deane, Newton, Deary, Rapley, Ng.

### Acquisition of data

Hackett, Newton, Deary, Bowman, Ng.

### Analysis and interpretation of data

Hackett, Deane, Newton, Deary, Rapley, Ng.

## Supporting information

Supplementary Figure 1

Supplementary Figure 2

Supplementary Figure 3

## References

[1] Fox RI . Sjögren's syndrome. Lancet 2005;366:321–31. 16039337 10.1016/S0140-6736(05)66990-5

[2] Rischmueller M , Tieu J , Lester S . Primary Sjögren's syndrome. Best Pract Res Clin Rheumatol 2016;30:189–220. 27421224 10.1016/j.berh.2016.04.003

[3] Segal BM , Pogatchnik B , Henn L , Rudser K , Sivils KM . Pain severity and neuropathic pain symptoms in primary Sjögren's syndrome: a comparison study of seropositive and seronegative Sjogren's syndrome patients. Arthritis Care Res (Hoboken) 2013;65:1291–8. 23335582 10.1002/acr.21956 PMC4137866

[4] Hackett KL , Gotts Z , Ellis J , Deary V , Rapley T , Ng WF , et al. An investigation into the prevalence of sleep disturbances in primary Sjögren's syndrome: a systematic review of the literature. Rheumatology (Oxford) 2017;56:570–80. 28013207 10.1093/rheumatology/kew443 PMC5410987

[5] Ng WF , Bowman SJ . Primary Sjögren's syndrome: too dry and too tired. Rheumatology (Oxford) 2010;49:844–53. 20147445 10.1093/rheumatology/keq009

[6] Valtysdottir ST , Gudbjornsson B , Lindqvist U , Hallgren R , Hetta J . Anxiety and depression in patients with primary Sjögren's syndrome. J Rheumatol 2000;27:165–9. 10648034

[7] Lendrem D , Mitchell S , McMeekin P , Bowman S , Price E , Pease CT , et al. Health‐related utility values of patients with primary Sjögren's syndrome and its predictors. Ann Rheum Dis 2014;73:1362–8. 23761688 10.1136/annrheumdis-2012-202863

[8] Cornec D , Devauchelle‐Pensec V , Mariette X , Jousse‐Joulin S , Berthelot JM , Perdriger A , et al. Severe health‐related quality‐of‐life impairment in active primary Sjögren's syndrome and patient‐reported outcomes: data from a large therapeutic trial. Arthritis Care Res (Hoboken) 2017;69:528–35. 27390310 10.1002/acr.22974

[9] Hackett KL , Newton JL , Frith J , Elliott C , Lendrem D , Foggo H , et al. Impaired functional status in primary Sjögren's syndrome. Arthritis Care Res (Hoboken) 2012;64:1760–4. 23111856 10.1002/acr.21738

[10] Hackett KL , Newton JL , Ng WF . Occupational therapy: A potentially valuable intervention for people with primary Sjögren's syndrome. Br J Occup Ther 2012;75:247–9.

[11] Priori R , Minniti A , Derme M , Antonazzo B , Brancatisano F , Ghirini S , et al. Quality of sexual life in women with primary Sjögren's syndrome. J Rheumatol 2015;42:1427–31. 26136488 10.3899/jrheum.141475

[12] Westhoff G , Dorner T , Zink A . Fatigue and depression predict physician visits and work disability in women with primary Sjögren's syndrome: results from a cohort study. Rheumatology (Oxford) 2012;51:262–9. 21705778 10.1093/rheumatology/ker208

[13] Ramos‐Casals M , Tzioufas AG , Stone JH , Sisó A , Bosch X . Treatment of primary Sjögren syndrome: a systematic review. JAMA 2010;304:452–60. 20664046 10.1001/jama.2010.1014

[14] Brito‐Zeron P , Baldini C , Bootsma H , Bowman SJ , Jonsson R , Mariette X , et al. Sjögren syndrome. Nat Rev Dis Primers 2016;2:16047. 27383445 10.1038/nrdp.2016.47

[15] Gottenberg JE , Ravaud P , Puechal X , Le Guern V , Sibilia J , Goeb V , et al. Effects of hydroxychloroquine on symptomatic improvement in primary Sjögren syndrome: the JOQUER randomized clinical trial. JAMA 2014;312:249–58. 25027140 10.1001/jama.2014.7682

[16] Devauchelle‐Pensec V , Mariette X , Jousse‐Joulin S , Berthelot JM , Perdriger A , Puechal X , et al. Treatment of primary Sjögren syndrome with rituximab: a randomized trial. Ann Intern Med 2014;160:233–42. 24727841 10.7326/M13-1085

[17] Bowman SJ , Everett CC , O'Dwyer JL , Emery P , Pitzalis C , Ng WF , et al. Randomized controlled trial of rituximab and cost‐effectiveness analysis in treating fatigue and oral dryness in primary Sjögren's syndrome. Arthritis Rheumatol 2017;69:1440–50. 28296257 10.1002/art.40093

[18] Kim B , Lucie L . Designing active communities: a coordinated action framework for planners and public health professionals. J Phys Act Health 2014;11:1041–51. 23492983 10.1123/jpah.2012-0178

[19] Trochim W . An introduction to concept mapping for planning and evaluation. Eval Program Plann 1989;12:1–16.

[20] Selten EM , Geenen R , van der Laan WH , van der Meulen‐Dilling RG , Schers HJ , Nijhof MW , et al. Hierarchical structure and importance of patients’ reasons for treatment choices in knee and hip osteoarthritis: a concept mapping study. Rheumatology (Oxford) 2017;56:271–8. 27864564 10.1093/rheumatology/kew409

[21] Trudeau KJ , Ainscough JL , Pujol LA , Charity S . What arthritis pain practitioners and patients want in an online self‐management programme. Musculoskeletal Care 2010;8:189–96. 21108492 10.1002/msc.183 PMC3058299

[22] Varekamp I , Haafkens JA , Detaille SI , Tak PP , van Dijk FJ . Preventing work disability among employees with rheumatoid arthritis: what medical professionals can learn from the patients’ perspective. Arthritis Rheum 2005;53:965–72. 16342108 10.1002/art.21592

[23] Hackett KL , Lambson RL , Strassheim V , Gotts Z , Deary V , Newton JL . A concept mapping study evaluating the UK's first NHS generic fatigue clinic. Health Expect 2016;19:1138–49. 26332418 10.1111/hex.12405 PMC5054859

[24] Kane M , Trochim WM . Concept mapping for planning and evaluation. London: Sage; 2007.

[25] Hackett KL , Newton JL , Deane KH , Rapley T , Deary V , Kolehmainen N , et al. Developing a service user informed intervention to improve participation and ability to perform daily activities in primary Sjögren's syndrome: a mixed‐methods study protocol. BMJ Open 2014;4:e006264. 10.1136/bmjopen-2014-006264 PMC4156812 25146718

[26] Ng WF , Bowman SJ , Griffiths B . United Kingdom Primary Sjögren's Syndrome Registry: a united effort to tackle an orphan rheumatic disease. Rheumatology (Oxford) 2011;50:32–9. 20693261 10.1093/rheumatology/keq240

[27] Vitali C , Bombardieri S , Jonsson R , Moutsopoulos HM , Alexander EL , Carsons SE , et al. Classification criteria for Sjögren's syndrome: a revised version of the European criteria proposed by the American‐European Consensus Group. Ann Rheum Dis 2002;61:554–8. 12006334 10.1136/ard.61.6.554 PMC1754137

[28] Zigmond AS , Snaith RP . The Hospital Anxiety and Depression Scale. Acta Psychiatr Scand 1983;67:361–70. 6880820 10.1111/j.1600-0447.1983.tb09716.x

[29] Broadbent DE , Cooper PF , FitzGerald P , Parkes KR . The Cognitive Failures Questionnaire (CFQ) and its correlates. Br J Clin Psychol 1982;21 Pt 1:1–16. 7126941 10.1111/j.2044-8260.1982.tb01421.x

[30] Fries JF , Cella D , Rose M , Krishnan E , Bruce B . Progress in assessing physical function in arthritis: PROMIS short forms and computerized adaptive testing. J Rheumatol 2009;36:2061–6. 19738214 10.3899/jrheum.090358

[31] Robinson BC . Validation of a Caregiver Strain Index. J Gerontol 1983;38:344–8. 6841931 10.1093/geronj/38.3.344

[32] De Kok M , Scholte RW , Sixma HJ , van der Weijden T , Spijkers KF , van de Velde CJ , et al. The patient's perspective of the quality of breast cancer care: the development of an instrument to measure quality of care through focus groups and concept mapping with breast cancer patients. Eur J Cancer 2007;43:1257–64. 17467266 10.1016/j.ejca.2007.03.012

[33] Kruskal JB . Multidimensional scaling by optimizing goodness of fit to a nonmetric hypothesis. Psychometrika 1964;29:1–27.

[34] Lackner A , Ficjan A , Stradner MH , Hermann J , Unger J , Stamm T , et al. It's more than dryness and fatigue: the patient perspective on health‐related quality of life in primary Sjögren's syndrome: a qualitative study. PLoS One 2017;12:e0172056. 28182787 10.1371/journal.pone.0172056 PMC5300216

[35] European Patients Forum . EPF background brief: patient empowerment. Brussels: EPF; 2015.

[36] Bravo P , Edwards A , Barr PJ , Scholl I , Elwyn G , McAllister M , et al. Conceptualising patient empowerment: a mixed methods study. BMC Health Serv Res 2015;15:252. 26126998 10.1186/s12913-015-0907-z PMC4488113

[37] Abourazzak F , El Mansouri L , Huchet D , Lozac'hmeur R , Hajjaj‐Hassouni N , Ingels A , et al. Long‐term effects of therapeutic education for patients with rheumatoid arthritis. Joint Bone Spine 2009;76:648–53. 19775924 10.1016/j.jbspin.2009.01.010

[38] Strombeck B , Jacobsson LT . The role of exercise in the rehabilitation of patients with systemic lupus erythematosus and patients with primary Sjögren's syndrome. Curr Opin Rheumatol 2007;19:197–203. 17278938 10.1097/BOR.0b013e32801494e3

[39] Strombeck BE , Theander E , Jacobsson LT . Effects of exercise on aerobic capacity and fatigue in women with primary Sjögren's syndrome. Rheumatology (Oxford) 2007;46: 868–71. 17308315 10.1093/rheumatology/kem004

[40] Anderson DR , Zlateva I , Coman EN , Khatri K , Tian T , Kerns RD . Improving pain care through implementation of the Stepped Care Model at a multisite community health center. J Pain Res 2016;9:1021–9. 27881926 10.2147/JPR.S117885 PMC5115680

[41] Davies B , Cramp F , Gauntlett‐Gilbert J , Wynick D , McCabe CS . The role of physical activity and psychological coping strategies in the management of painful diabetic neuropathy: a systematic review of the literature. Physiotherapy 2015;101:319–26. 26036692 10.1016/j.physio.2015.04.003

[42] Department of Health and Social Care . Personalised health and care 2020: a framework for action. 2014 URL: https://www.gov.uk/government/publications/personalised-health-and-care-2020/using-data-and-technology-to-transform-outcomes-for-patients-and-citizens.

[43] Priori R , Iannuccelli C , Alessandri C , Modesti M , Antonazzo B , Di Lollo AC , et al. Fatigue in Sjogren's syndrome: relationship with fibromyalgia, clinical and biologic features. Clin Exp Rheumatol 2010;28 Suppl 63:S82–6. 21176426

[44] Choi BY , Oh HJ , Lee YJ , Song YW . Prevalence and clinical impact of fibromyalgia in patients with primary Sjögren's syndrome. Clin Exp Rheumatol 2016;34 Suppl 96:S9–13. 26315451

[45] Singh AG , Singh S , Matteson EL . Rate, risk factors and causes of mortality in patients with Sjögren's syndrome: a systematic review and meta‐analysis of cohort studies. Rheumatology (Oxford) 2016;55:450–60. 26412810 10.1093/rheumatology/kev354 PMC5009445

