# Supplementary material for: Mixed‐Methods Study Identifying Key Intervention Targets to Improve Participation in Daily Living Activities in Primary Sjögren's Syndrome Patients
Source: Arthritis Care Res (Hoboken). 2018 May 21;70(7):1064–73. doi: 10.1002/acr.23536 (PMC6033158; doi:10.1002/acr.23536)

Supplementary Figure 1: Flow diagrams demonstrating recruitment of participants through the study

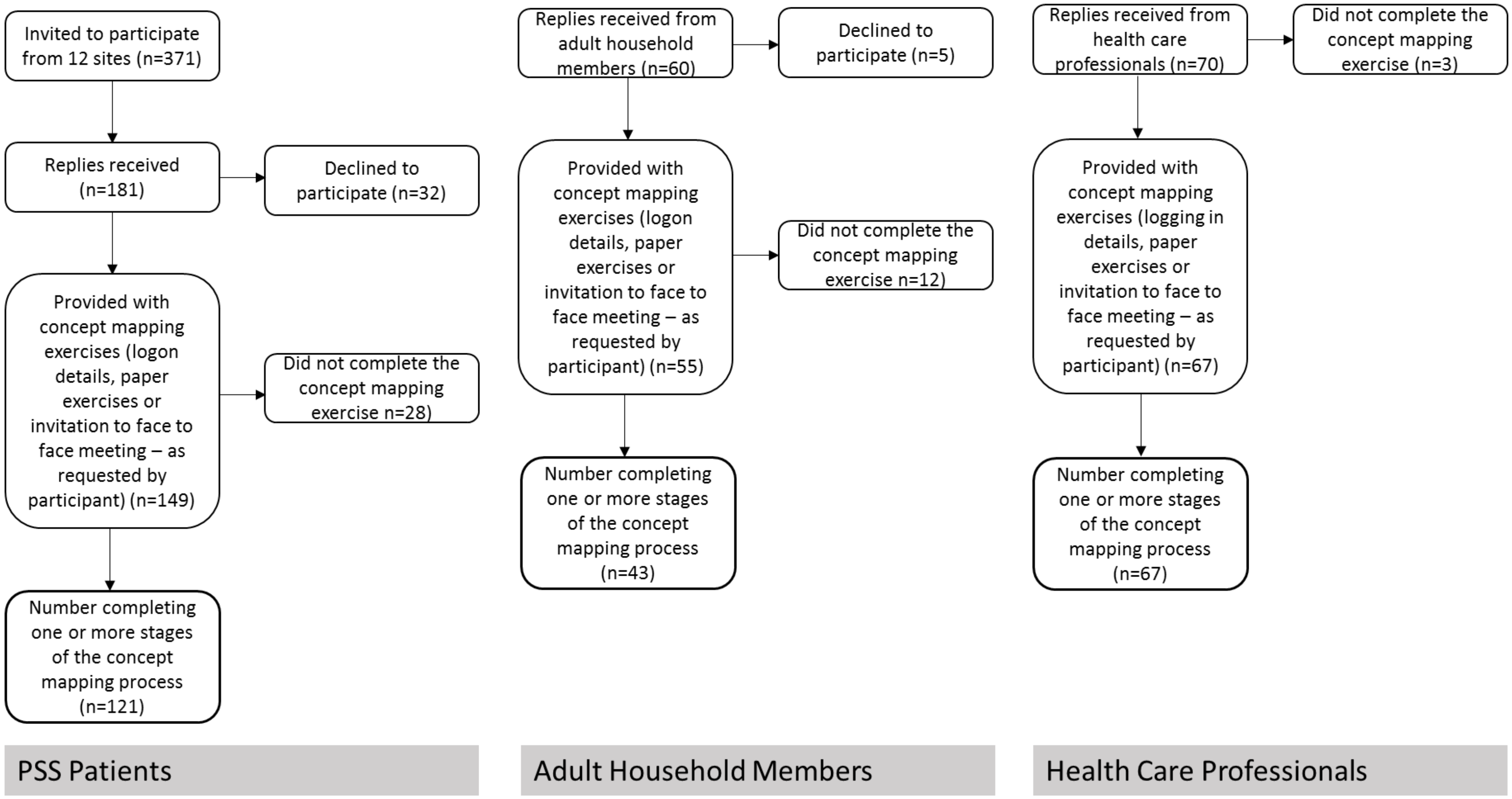

Supplement: Supplementary file 1 — Supplementary Figure 1 [file ACR-70-1064-s001.pdf]
