# Supplementary material for: Mixed‐Methods Study Identifying Key Intervention Targets to Improve Participation in Daily Living Activities in Primary Sjögren's Syndrome Patients
Source: Arthritis Care Res (Hoboken). 2018 May 21;70(7):1064–73. doi: 10.1002/acr.23536 (PMC6033158; doi:10.1002/acr.23536)

Supplementary Figure 2: Breakdown of professional groups within the health care professional participants

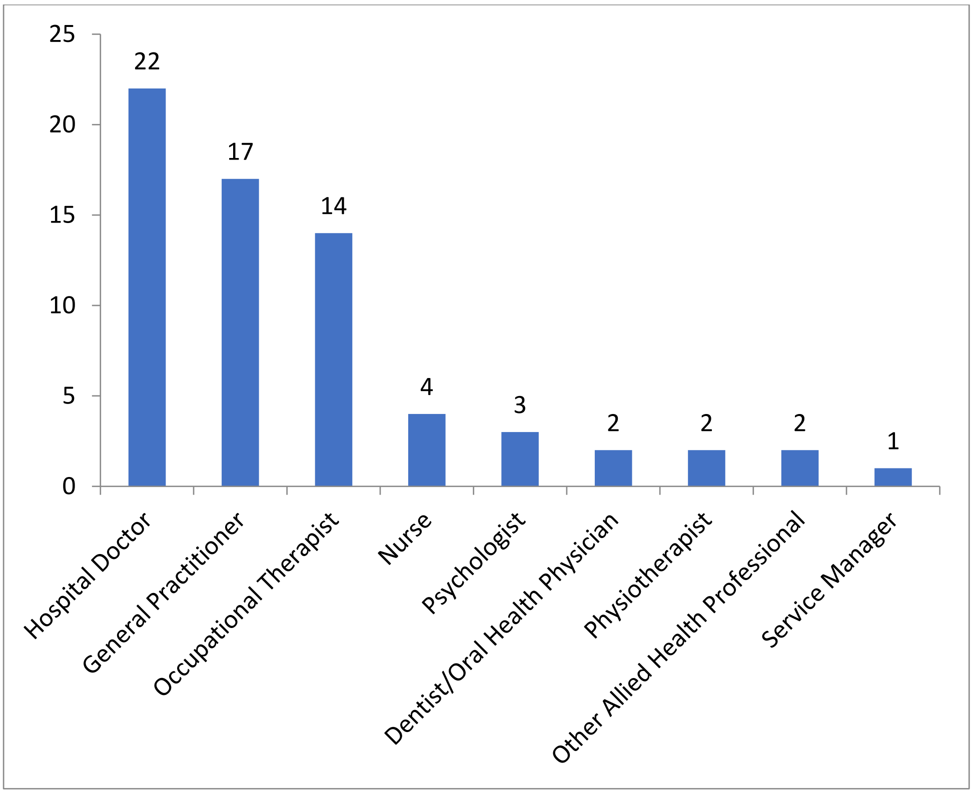

Supplement: Supplementary file 2 — Supplementary Figure 2 [file ACR-70-1064-s002.pdf]
