# Supplementary material for: Mixed‐Methods Study Identifying Key Intervention Targets to Improve Participation in Daily Living Activities in Primary Sjögren's Syndrome Patients
Source: Arthritis Care Res (Hoboken). 2018 May 21;70(7):1064–73. doi: 10.1002/acr.23536 (PMC6033158; doi:10.1002/acr.23536)
Supplement: Supplementary file 3 — Supplementary Figure 3 [file ACR-70-1064-s003.pdf]

Supplementary Figure 3: Go-zones showing the most important statements within the lower priority clusters

Knowledge & support

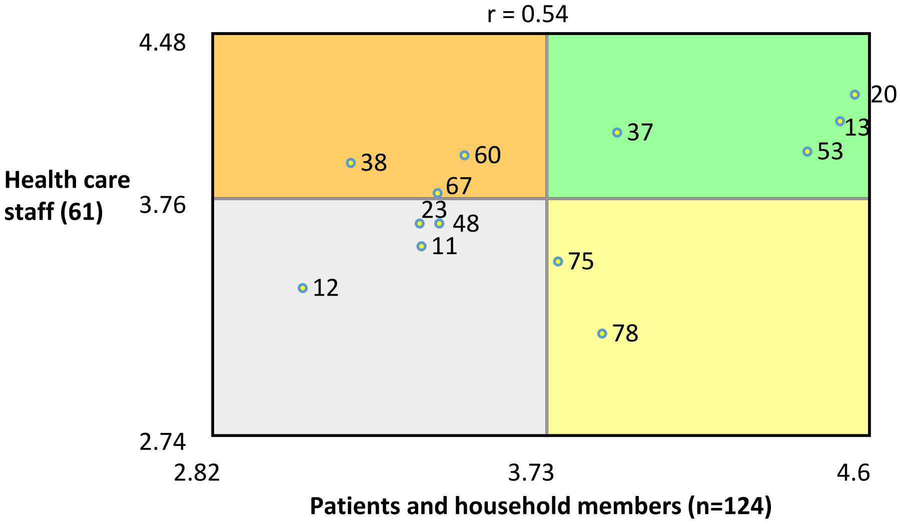

13. There was more good research to understand the underlying causes
20. There was more good research to test and develop treatments
37. There was education on Sjogren's for patients
53. There was education on Sjogren's for healthcare professionals

Friends & family

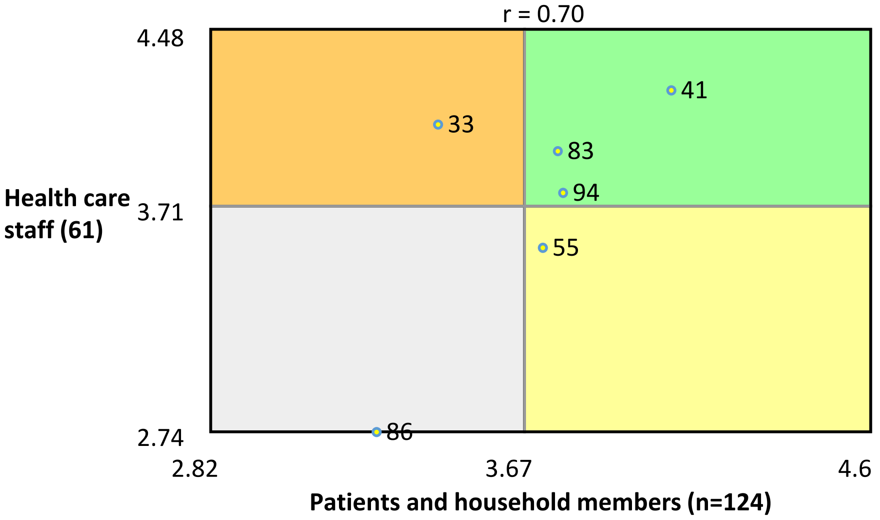

41. Have supportive family and friends
83. Family could understand the symptoms
94. Can explain to others what they can and cannot do

Public awareness & support

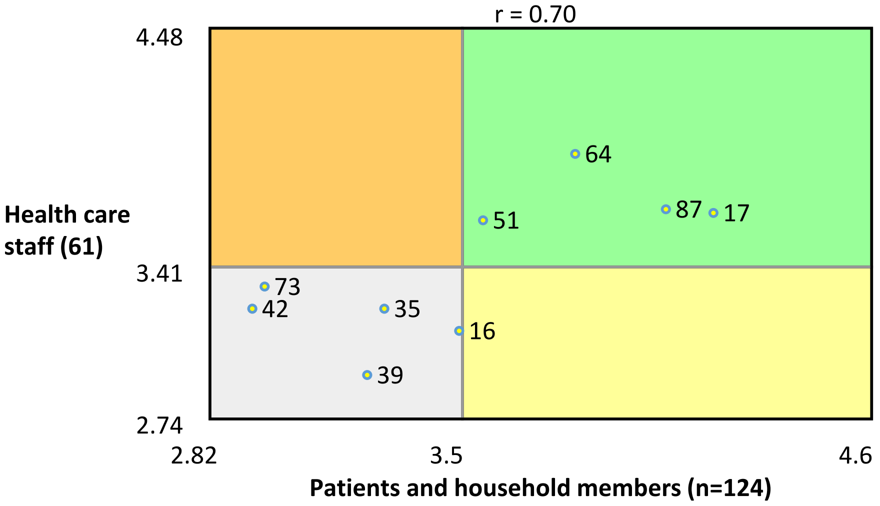

17. There was education about Sjogren's for people who fund services
51. There was education on Sjogren's for family members
64. Employers were aware of things they could do in the workplace that are helpful for people with Sjogren's
87. Those unable to work and/or needed support to function, were eligible for benefits
